# Enhancing practical utility and policy integration of ultra-processed food screeners

**DOI:** 10.1017/S1368980026102900

**Published:** 2026-06-05

**Authors:** Kervin Blanco Untalan, Genessa Jesy Teroy Pagote, Kyle Renz Gomez Tejero

**Affiliations:** 1 Food Technology, Bukidnon State University, Philippines; 2 Natural Science, Bukidnon State University, Philippines

Dear Editor,

Hamel *et al.* ^(1)^ present an important contribution through the development and validation of the Canadian ultra-processed product screener, demonstrating promising construct validity and reliability within the NOVA classification framework. The advancement of brief and scalable dietary assessment tools is critical for monitoring ultra-processed food consumption and informing public health strategies targeting diet-related non-communicable diseases. However, several methodological and contextual considerations warrant further refinement to strengthen the broader applicability and policy relevance of the instrument.

First, reliance on self-reported dietary frequency introduces inherent limitations, including recall bias and systematic misreporting, particularly for foods perceived as unhealthy. Recent evidence suggests that while technology-assisted dietary tools can enhance feasibility and user engagement, improvements in accuracy remain inconsistent^(2)^. A pragmatic approach may involve combining simplified recall strategies with selective integration of digital tools to improve data quality without increasing respondent burden.

Second, the screener may benefit from incorporating food environment dimensions. Dietary behaviours are shaped not only by individual choices but also by structural determinants such as food availability, affordability and marketing exposure. Contemporary analyses emphasise the strong influence of food systems and commercial determinants on UPF consumption patterns^(3)^. Integrating simple contextual indicators (e.g. food source or perceived affordability) could enhance construct validity while maintaining feasibility.

Third, while the NOVA classification provides a widely used framework, concerns regarding its operational consistency persist. Recent discussions highlight ambiguities in classification boundaries that may affect comparability across studies^(4)^. Complementing NOVA-based categorisation with standardised examples or selected nutrient-profile indicators may improve interpretability and consistency.

From a translational perspective, embedding the screener into existing public health systems offers a realistic pathway for impact. Brief dietary assessment tools can support surveillance and early identification of dietary risks. Incorporating automated feedback mechanisms, such as risk categorisation with basic dietary guidance, may allow the screener to function as both an assessment and intervention tool.

Finally, expanding the applicability of the screener to low- and middle-income countries remains essential. UPF consumption continues to rise globally, contributing to the double burden of malnutrition^(5)^. Context-specific adaptation, including culturally relevant food lists and locally validated consumption patterns, will be necessary to ensure external validity and global relevance.

In conclusion, the work of Hamel *et al.* ^(1)^ represents a significant advancement in rapid dietary assessment of ultra-processed food consumption. Its utility can be further strengthened through feasible methodological refinements, integration of environmental context and alignment with public health systems, thereby enhancing its contribution to equitable and evidence-based nutrition policy.
